# Comparison of QIAstat-Dx and BioFire FilmArray Gastrointestinal Panels in a Pediatric Population

**DOI:** 10.3390/microorganisms12112282

**Published:** 2024-11-10

**Authors:** Mohammed Suleiman, Muhammad Iqbal, Patrick Tang, Andrés Pérez-López

**Affiliations:** 1 Department of Pathology, Sidra Medicine, Doha P.O. Box 26999, Qatar; miqbal@sidra.org (M.I.); ptang@sidra.org (P.T.); aperezlopez@sidra.org (A.P.-L.); 2 Department of Pathology and Laboratory Medicine, Weill Cornell Medicine in Qatar, Doha P.O. Box 24144, Qatar

**Keywords:** GI panel, syndromic testing, QIAstat, BioFire

## Abstract

Accurate laboratory diagnosis of gastroenteritis is important to ensure that patients receive appropriate treatment and proper isolation precautions. This study evaluated the performance of the QIAGEN QIAstat-Dx gastrointestinal panel (QGP) in comparison to the bioMerieux BioFire FilmArray gastrointestinal panel (BGP) for the detection of gastrointestinal pathogens in 110 pediatric patients being evaluated for gastroenteritis at our hospital. We compared 23 different bacterial, viral, and parasite enteropathogens detected by the QGP against the BGP. The overall positive percent agreement (PPA) for all compared targets was 96.2% and the overall negative percent agreement (NPA) for all compared targets was 99.7%. Our study shows that QIAstat-Dx QGP provides comparable results to the BioFire BGP in our pediatric population. Additionally, the PCR cycle threshold (Ct) value reported by the QGP is potentially a helpful tool in estimating the load of the detected pathogen in stool samples.

## 1. Introduction

Gastroenteritis is an inflammation of the gastrointestinal tract, leading to many symptoms including nausea, vomiting, cramping, abdominal pain, and diarrhea [1]. It can be caused by a variety of pathogens including viruses, bacteria, and parasites. Most cases are transient and self-limiting, but, in some cases, it can lead to severe illness and hospitalization. Globally, gastroenteritis remains a significant public health challenge and is one of leading causes of death in children younger than five years old, killing an estimated 370,000 children in 2019 [2]. 

Microbiological diagnosis is essential to guide clinical management, including the decision to treat with antimicrobials or not, and to ensure that patients are placed on appropriate isolation precautions. The laboratory diagnosis of gastroenteritis has recently evolved from standard microbiological methods, which include stool culture, an antigen test, and an microscopic exam for ova and parasites, to more sensitive, specific, and faster multiplex PCR panels [3,4,5]. Several studies have shown that these multiplex PCR panels have the potential to improve clinical management and lower healthcare costs, including the reduction in antibiotic consumption, length of hospital stay, and diagnostic testing [3,6,7]. 

In this study, we evaluated the performance of a new multiplex PCR panel, the QIAGEN QIAstat-Dx gastrointestinal panel (QGP; Hilden, Germany), against the performance of the bioMerieux BioFire FilmArray gastrointestinal panel (BGP; bioMerieux, Marcy l’Étoile, France) for the detection of GI pathogens using clinical stool samples from our pediatric population at Sidra Medicine. 

## 2. Materials and Methods 

### 2.1. Specimen Collection and Study Design 

This study was approved by the Institutional Review Board at Sidra Medicine (Project number 20424901). Stool samples were prospectively collected from 110 pediatric patients being evaluated for symptomatic gastroenteritis. Samples were received from inpatient units, outpatient clinics, and the emergency department at Sidra Medicine, a tertiary care pediatric reference center in Doha, Qatar. Samples were collected in sterile containers between October 2023 and November 2023. The study included all samples that were received within the study period that had sufficient quantity. Samples were transferred to Cary–Blair medium and tested immediately upon receipt in our laboratory. All samples were tested using the QGP and BGP methods according to the manufacturer’s instructions by licensed clinical laboratory staff. 

### 2.2. QGP Method 

The QGP is a qualitative test that detects nucleic acid from twenty-three common bacterial, viral, and parasitic pathogens collected from patients with gastroenteritis (Table 1). Version 2 of the QGP assay was used in our study. This assay is used with the QIAstat-Dx Analyzer (QIAGEN, Hilden, Germany), which is an automated instrument that integrates nucleic acid extraction with multiplex real-time PCR detection. The sample is vortexed and 200 μL of the diluted mixture is transferred into the QGP test cartridge. The cartridge is closed and loaded into the instrument. The test run time is 76 min and the software provides a report for all twenty-three targets and for the internal control. For samples with any targets detected, the PCR cycle threshold (Ct value) and amplification curves are available on the printed report.

### 2.3. BGP Method 

The BGP is also a qualitative test that detects nucleic acid from twenty-two common bacterial, viral, and parasitic pathogens collected from patients with gastroenteritis (Table 1). The assay is used with the Biofire Filmarray Torch system (bioMerieux, Marcy l’Étoile, France), which is an automated instrument that integrates nucleic acid extraction with nested multiplex PCR detection. The test run time is about one hour and the software provides a report for all twenty-two targets. The Biofire system is based on endpoint detection of PCR targets and therefore does not provide a PCR Ct value for the detected targets. 

### 2.4. Statistical Analysis 

To evaluate the performance of QGP in comparison to the BGP method, false positive (FP), true positive (TP), false negative (FN), and true negative (TN) status was assigned to each of the targets detected by the QGP with the BGP results serving as the reference as there was no third method available to resolve discrepancies. In addition, positive percent agreement (PPA) and negative percent agreement (NPA) were calculated for each of the targets detected by the QGP. PPA was calculated as (TP/TP  +  FN) × 100%  and NPA was calculated as (TN/TN  +  FP) × 100%. 

## 3. Results 

### 3.1. Samples and Demographics 

The mean patient age was 6.9 years old (range 0–18), 48% of patients were younger than 6 years old, 33.6% were between 6 and 12 years old, and 18.4% between 12 and 18 years old. Sixty-three (57.2%) samples were collected from male patients while forty-seven (43.8%) samples were collected from female patients. Forty-eight (43.6%) samples originated from inpatient units and sixty-two (56.4%) came from outpatient clinics and the emergency room.

### 3.2. Summary of Findings 

Of the 110 stool samples evaluated, 98 (89.1%) had fully concordant results between both methods and 69 were positive by both tested methods (positive for at least one target), as shown in Table 2. A total of 29 out of 110 (26.3%) samples tested by the BGP had multiple pathogens (two to four pathogens) while 23 out of the 110 (20.9%) samples tested by the QGP had multiple pathogens (two to three pathogens). Table 2 shows the number of QGP and BGP positive samples by the number of detected pathogens. 

The PPA and NPA for each target detected on the QGP in comparison to the BGP is shown in Table 3. The overall positive percent agreement (PPA) for all compared targets was 96.2% and the overall negative percent agreement (NPA) for all compared targets was 99.7%. Twelve (10.9%) samples had discordant results between the QGP and BGP. Four samples had a positive target detected only by QGP while eight samples had a positive target detected only by the BGP. Three discordant samples had one pathogen detected in one method only. The remaining nine samples had only one discrepant target from the multiple (2–4) targets detected in each sample. Out of the 23 samples that had two or three pathogens detected using the QGP, a single dominant pathogen could be identified in 16 samples by applying the criteria of a strong Ct value <27 or a Ct value difference in greater than four cycles. In 14 of those 16 samples, the comparisons involved only single copy gene targets. However, in two of the sixteen samples, the dominant pathogen was detected through a multi-copy gene but in each case, the Ct difference was greater than 7. In addition, in two samples, more than one pathogen was detected using the QGP with a Ct value below 27, and the Ct value difference between the targets was less than 4, suggesting that more than one of the targets may have been significant.

## 4. Discussion

Our study suggests that QGP provides comparable results to BGP based on the analysis of the targets found on both panels. To our knowledge, this is the first study comparing the performance of QGP to BGP in a pediatric population and only the second study to evaluate the second version of the QGP. There are few studies currently available in the literature evaluating the performance of the QGP assay. One study which evaluated the first version of the QGP against BGP had similar results to our study [3]. They had fully concordant results in 345 (89.6%) out of 385 samples and an overall PPA 98.2% and an NPA of 99.9%, similar to the results of our study [3]. Another study compared the performance of the first version of the QGP to a laboratory-developed PCR method and found an overall agreement of 95% when they excluded discrepant results with a Ct value above 35 [8]. A study that evaluated the second version of the QGP compared it against the Luminex xTAG gastrointestinal pathogen panel (Luminex Molecular Diagnostics, Austin, TX, USA) for 10 targets only and showed an excellent correlation with an NPA of 98.9 and an PPA of 91–100% [9].

Several other studies compared the performance of other commercially available multiplex PCR panels for the detection of gastroenteritis. One major study evaluated six FDA-cleared assays, including BGP, Luminex xTAG panel, Verigene Enteric Pathogens Nucleic Acid Test (Luminex Molecular Diagnostics, Austin, TX, USA), Applied BioCode Gastrointestinal Pathogen Panel (BioCode, Santa Fe Springs, CA, USA), Prodesse ProGastro SSCS Assay (Hologic, Marlborough, MA, USA), and EntericBio Dx Gastrointestinal Panel (Quidel, San Diego, CA, USA) [10]. All assays in this study were able to detect most of the tested pathogens and had an excellent specificity of 98–100% [10].

A major advantage for the use of the QGP assay over some of other commercially available assays such as the BGP and Luminex xTAG panel is the ability to generate Ct values for the detected targets to provide an indication of the relative pathogen load. This can be helpful in the interpretation of results when multiple pathogens are detected on the panel. In samples with multiple pathogens, the ones with a stronger Ct value may be more likely to be the cause of the infection with consideration for the average copy number of each PCR target. Three discordant samples in our study had Ct values between 34.0 and 37.8, suggesting that these targets were in low abundance in the specimen and may not be reproducible by the BGP. The correlation between the Ct value and organism concentration has not been well studied in multiplex Gastrointestinal PCR panels, and more comprehensive clinical and laboratory studies are required to determine the role of Ct values in the diagnosis of infectious gastroenteritis. In a previous study on respiratory infections, the promising results showed a potential role for using the Ct values provided by the QIAstat Respiratory SAR-CoV-S panel as a surrogate for viral load in the samples [11].

Our study had several limitations. First, the performance of the QGP assay to detect rotavirus, *Plesiomonas shigelloides*, shiga-like toxin-producing *E. coli* (STEC), *E. coli* O157 serogroup, *Cyclospora cayetanensis*, and *Vibrio* species could not be assessed due to a lack of positive fecal samples for these targets during the time of our study. The absence of positive samples for certain pathogens in the study limits the ability to fully assess the comparative performance of the QGP and BGP for those specific targets. Second, our laboratory did not have a third independent method to resolve discrepant samples. The majority of the discrepancies were in samples with diarrheagenic *E. coli* organisms (EPEC, ETEC, EAEC, EIEC). Although they are commonly detected (mostly in co-infections) in the multiplex PCR panels, the clinical relevance and pathogenicity of these organisms remain unclear and requires further research [8,12,13]. The lack of a third method to resolve the discordant results could have biased the results in favor of the BGP and reduced the accuracy of the QGP method. 

In conclusion, the QGP performed well for the molecular diagnosis of gastroenteritis with excellent PPA and NPA against the BGP in our pediatric population. The Ct value provided by the QGP also offers a potential advantage over the BGP and could be helpful in estimating the relative abundance of each pathogen in positive samples. However, further clinical studies of the QGP Ct values would be required to understand their additional value in the diagnosis and management of gastroenteritis.

## Figures and Tables

**Table 1:** Comparison of targets detected on BGP and QGP.

	BGP	QGP
Number of Targets	22	23
Bacteria	*Campylobacter* (*C. jejuni*/*C. coli*/*C. upsaliensis*)	*Campylobacter* (*C. jejuni*/*C. coli*/*C. upsaliensis*)
	*Clostridium difficile* (toxin A/B)	*Clostridium difficile* (toxin A/B)
	*Plesiomonas shigelloides*	*Plesiomonas shigelloides*
	*Salmonella* spp.	*Salmonella* spp.
	*Vibrio* (*V. parahaemolyticus*/*V. vulnificus*/*V. cholerae*) *	Vibrio vulnificus *
		Vibrio parahaemolyticus *
	*Vibrio cholerae*	*Vibrio cholerae*
	*Yersinia enterocolitica*	*Yersinia enterocolitica*
	Enteroaggregative *E. coli* (EAEC)	Enteroaggregative *E. coli* (EAEC)
	Enteropathogenic *E. coli* (EPEC)	Enteropathogenic *E. coli* (EPEC)
	Enterotoxigenic *E. coli* (ETEC) lt/st	Enterotoxigenic *E. coli* (ETEC) lt/st
	Shiga-like toxin-producing *E. coli* (STEC) stx1/stx2	Shiga-like toxin-producing *E. coli* (STEC) stx1/stx2
	*E. coli* O157	*E. coli* O157
	*Shigella*/Enteroinvasive *E. coli* (EIEC)	*Shigella*/Enteroinvasive *E. coli* (EIEC)
Viruses	Adenovirus F40/F41	Adenovirus F40/F41
	Astrovirus	Astrovirus
	Norovirus GI/GII	Norovirus GI/GII
	Rotavirus A	Rotavirus A
	Sapovirus (GI, GII, GIV, and GV)	Sapovirus (GI, GII, GIV, and GV)
Parasites	*Cryptosporidium*	*Cryptosporidium*
	*Cyclospora cayetanensis*	*Cyclospora cayetanensis*
	*Entamoeba histolytica*	*Entamoeba histolytica*
	*Giardia lamblia*	*Giardia lamblia*

* The main difference between both panels is that the QGP differentiates *Vibrio vulnificus* from *Vibrio parahaemolyticus*. BGP, BioFire FilmArray gastrointestinal panel; QGP, QIAstat-Dx Gastrointestinal Panel 2 PCR test.

**Table 2:** Number of pathogens detected in each specimen by each method.

	No. of Specimens (%)		% of Positive Specimens	
No. of Pathogens Detected	QGP	BGP	QGP	BGP
0	40 (36.30%)	41 (37.30%)	NA	NA
1	47 (42.70%)	40 (36.30%)	67.10%	58%
2	12 (11.00%)	20 (18.20%)	17.10%	29%
3	11 (10.00%)	8 (7.30%)	15.80%	11.60%
4	0	1 (0.90%)	0	1.40%

BGP, BioFire FilmArray gastrointestinal panel; NA, not applicable; QGP, QIAstat-Dx Gastrointestinal Panel 2 PCR test.

**Table 3:** Performance summary of QGP in comparison to BGP.

Pathogen	FN	FP	TP	TN	PPA (95% CI)	NPA (95% CI)
*Campylobacter*	0	0	3	107	100 (44–100%)	100 (97–100%)
*Clostridioides difficile*	1	0	23	86	100 (86–100%)	99 (94–100%)
*Plesiomonas shigelloides*	0	0	0	110	Not applicable	100 (97–100%)
*Salmonella*	0	0	10	100	100 (72–100%)	100 (96–100%)
*Yersinia enterocolitica*	0	1	0	109	Not applicable	100 (97–100%)
*Vibrio cholera*	0	0	0	110	Not applicable	100 (97–100%)
*Vibrio parahaemolyticus*	0	0	0	110	Not applicable	100 (97–100%)
*Vibrio vulnificus*	0	0	0	110	Not applicable	100 (97–100%)
Enteroaggregative *E. coli* (EAEC)	1	1	13	95	93 (69–99%)	99 (94–100%)
Enteropathogenic *E. coli* (EPEC)	3	0	13	94	100 (77–100%)	97 (91–99%)
Enterotoxigenic *E. coli* (ETEC) lt/st	1	0	5	104	100 (57–100%)	99 (95–100%)
Shiga-like toxin-producing *E. coli* (STEC)	0	0	0	110	Not applicable	100 (97–100%)
*E. coli* O157 serogroup	0	0	0	110	Not applicable	100 (97–100%)
Shigella/enteroinvasive *E. coli* (EIEC)	0	1	4	105	80 (38–96%)	100 (96–100%)
*Cryptosporidium*	0	0	2	108	100 (34–100%)	100 (97–100%)
*Cyclospora cayetanensis*	0	0	0	110	Not applicable	100 (97–100%)
*Entamoeba histolytica*	0	0	1	109	100 (21–100%)	100 (97–100%)
*Giardia lamblia*	0	0	3	107	100 (44–100%)	100 (97–100%)
Adenovirus F40/41	0	0	6	104	100 (61–100%)	100 (96–100%)
Astrovirus	0	0	4	106	100 (51–100%)	100 (97–100%)
Norovirus GI/GII	1	1	5	103	83 (44–97%)	99 (95–100%)
Rotavirus A	0	0	0	110	Not applicable	100 (97–100%)
Sapovirus	1	0	9	100	100 (70–100%)	99 (95–100%)
Total	8	4	101	2417	96.2 (91–99%)	99.7 (99–100%)

BGP, BioFire FilmArray gastrointestinal panel; FN, false negative; FP, false positive; NPA, negative percent agreement; PPA, positive percent agreement; QGP, QIAstat-Dx Gastrointestinal Panel 2 PCR test; TN, true negative; TP, true positive.

## Data Availability

The datasets generated and/or analyzed during the current study are available from the corresponding author on reasonable request.
